# Family planning competency following medical school Ob/Gyn clerkships at faith-based and secular sites

**DOI:** 10.1038/s41598-024-54304-5

**Published:** 2024-02-14

**Authors:** Rachel N. Feltman, Steven R. Lewis, Nathan E. Thompson

**Affiliations:** 1 NYIT College of Osteopathic Medicine, Old Westbury, NY 11568 USA; 2 Department of Clinical Medicine, NYIT College of Osteopathic Medicine, Jonesboro, AR 72401 USA; 3 Department of Anatomy, NYIT College of Osteopathic Medicine, 100 Northern Boulevard, Riland 330, Old Westbury, NY 11568 USA

**Keywords:** Medical education, Abortion, Contraception, Obstetrics and gynecology, Clinical education, Clinical training, Health care, Health policy

## Abstract

Contraception and abortion topics are variably, but often poorly, addressed in medical school curricula. Restrictions on contraceptive and abortion care at faith-based hospitals may hinder comprehensive family planning training for medical students during Ob/Gyn clerkships. Here we investigated whether medical students at faith-based and non-faith-based clerkships experienced different observations during their Ob/Gyn clerkship and/or differences in self-perceived competency in patient counseling, objective knowledge, and perceived adequacy of training in contraception and abortion topics post-clerkship. A survey was distributed to third- and fourth-year medical students at New York Institute of Technology, College of Osteopathic Medicine. Across all clerkship sites (n = 102 students), observations of, and competency in, contraceptive care was higher than in abortion care. Students at non-faith-based clerkship sites (n = 54) reported the highest levels of observation of contraceptive and abortion care (19.6–90.7%), while those at Catholic sites (n = 26) typically reported the lowest (7.7–34.6%). Students at non-faith-based sites reported significantly higher competency in contraceptive care and some aspects of abortion care, than those at Catholic, and some other faith-based sites (n = 48). Clerkship training at faith-based sites, specifically Catholic sites, resulted in poorer Ob/Gyn training, particularly in contraceptive care. Training outcomes in abortion care were poor at all Ob/Gyn clerkship sites.

## Introduction

Family planning services, specifically contraception and abortion, are essential healthcare needs utilized by millions of patients each year. For many medical students, initial hands-on training in these topics occurs during their Ob/Gyn clerkship. While contraceptive and abortion care are included as educational objectives in nationwide guidelines for Ob/Gyn clerkships^1^, previous research has shown that these topics are poorly represented in Ob/Gyn curricula^2–6^.

One potential barrier to robust clerkship training is that many clerkships take place at faith-based hospitals or practices, some of which place restrictions on contraceptive and abortion care. In particular, Catholic-affiliated sites are expected to follow the “Ethical and Religious Directives for Catholic Healthcare Services” (ERDs)^7^. The ERDs place restrictions on the use of contraception and induced abortion procedures broadly across Catholic-affiliated hospitals and practices. Yet institutional objections and hospital-specific restrictions limiting family planning care have also been documented at faith-based hospitals of other religious denominations^8–10^. Together, this restrictive health care landscape likely affects the ability of Catholic and other faith-based institutions to provide comprehensive family planning training during clerkships. Previous research has shown that students at one Catholic medical school felt their clinical contraception and abortion training was overwhelmingly inadequate^6^ and standardized examination scores following the Ob/Gyn clerkship at another institution were lower for students at Catholic-affiliated sites^11^. These trends are also mirrored in residency programs, where many faith-based programs were found to be somewhat deficient in contraception training, and even more so in abortion training^12^.

Here, we evaluated whether medical students at New York Institute of Technology, College of Osteopathic Medicine (NYIT COM) who performed their Ob/Gyn clerkships at faith-based sites experienced differential outcomes compared to students at non-faith-based clerkship sites. NYIT COM is composed of two geographically separate sites (Old Westbury, New York and Jonesboro, Arkansas). Both sites follow the same educational curriculum and learning objectives, and at both sites Ob/Gyn clerkships take place at a mix of faith-based and non-faith-based sites. Specifically, regarding contraception and abortion training we evaluated if there was a difference in 1) what students observed during the Ob/Gyn clerkship, 2) self-perceived competency in patient counseling following the clerkship, 3) objective contraception and abortion knowledge, and 4) perceived adequacy of training received during the clerkship, between faith-based and non-faith-based sites. We hypothesized that students at faith-based clerkship sites, and in particular Catholic sites, would have lower outcomes in all four areas (1–4 above). Given the common Ob/Gyn educational curriculum, we did not expect to see any differences in clerkship outcomes between the two NYIT COM campus sites.

## Results

### Study demographics

E-mail survey invitations were sent to 845 eligible third- and fourth-year students, including two follow-up reminder emails. From this, 150 students opened the survey and 116 were returned completed (completion rate of 77.3% following CHERRIES guidelines^13^). Fourteen participants were excluded because they did not provide an identifiable clerkship site, resulting in a final total of 102 participants. Of these, 19 incorrectly reported, or did not know, the religious affiliation of their clerkship site. Following verification and correction, this resulted in a total of 28 clerkship sites represented in the sample (OW: 15; JB: 13) with 54 students at non-faith-based sites, 26 at Catholic sites, 15 at Jewish sites, and 7 at Protestant clerkship sites (Table 1). There were no significant associations between student religious identity or gender and the religious affiliation of the clerkship site they attended (Supplementary Table S1).

**Table 1:** Characteristics of survey respondents.

Affiliation of clerkship site	NFB	C	J	P	Total
Total students					
	54	26	15	7	102
Gender					
Female	34	17	10	6	67
Male	19	8	5	1	33
Prefer not to say/blank	1	1	–	–	2
Self-described racial identity					
East Asian	6	2	2	1	11
South Asian	10	5	2	1	18
Latino, Hispanic, or Spanish	3	2	–	1	6
Black or African American	2	0	2	0	4
Native Hawaiian or Pacific Islander	1	1	–	1	3
White	34	17	10	4	65
Prefer not to say	1	2	–	–	3
Religion					
Protestant	7	–	2	1	10
Catholic	7	10	5	1	23
Jewish	5	2	2	–	9
Muslim/Islam	5	3	1	–	9
Hindu	1	1	1	1	4
Other Christian	5	–	2	2	9
Other non-Christian religion	4	–	–	–	4
Not affiliated or none	20	10	2	2	34
Class year					
2023	19	7	6	6	38
2024	35	19	9	1	64
Campus site					
Jonesboro, Arkansas	9	4	–	7	20
Old Westbury, New York	45	22	15	–	82

NFB, non-faith-based; C, Catholic; J, Jewish; P, Protestant.

### Observations

**Figure 1:**
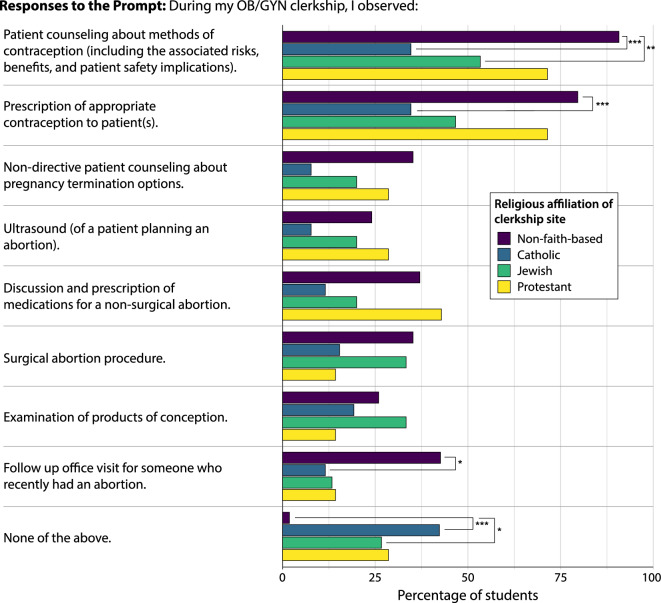
Results of student observations regarding family planning topics. All questions began with the prompt, “During my OB/GYN clerkship, I observed…”. All results are standardized by percentage of student responses for the religious affiliation of the clerkship site. Bars and asterisks represent a significant difference between religious affiliation of the clerkship (pairwise Fisher’s tests) at either the *P* < 0.05*, *P* < 0.01**, or *P* < 0.001*** level.

Across all sites students more frequently observed patient care related to contraception than any abortion-related care (Table 2, Fig. 1). Overall, 69.6% of students witnessed patient counseling on contraceptive methods and 62.7% observed prescription of contraception versus a maximum observation frequency of 28.4% for any abortion care topic. Among clerkship site affiliations, students at non-faith-based sites reported the highest levels of observation for most of the patient care topics presented (5 of 8). Students at Catholic sites reported the lowest levels of observation for 6 of 8 topics, and students at Jewish and Protestant sites tended to report intermediate levels of observation (Table 2; Fig. 1). Pairwise Fisher’s tests showed that students at non-faith-based sites observed significantly more contraceptive counseling compared to students at Catholic or Jewish sites (pairwise Fisher’s test: *P* < 0.001 and *P* < 0.01, respectively; Table 2) and significantly more prescribing of contraception compared to students at Catholic sites (pairwise Fisher’s test: *P* < 0.001). Students at non-faith-based sites also observed significantly more follow-up visits with a patient who recently had an abortion than students at Catholic sites (pairwise Fisher’s test: *P* < 0.05). Student observations of non-directive patient counseling about pregnancy termination options also showed a significant effect of clerkship site religious affiliation (Fisher’s exact test: *P* < 0.05), however no post-hoc pairwise tests reached the level of significance (Table 2). Only 1.9% of students at non-faith-based sites reported observing ‘none of the above’, significantly less than students at Catholic sites (42.3%, pairwise Fisher’s test: *P* < 0.001) and Jewish sites (26.7%, pairwise Fisher’s test: *P* < 0.05). Students at Protestant sites also reported moderate frequencies of ‘none of the above’ (28.6%), though this was not significantly different from students at non-faith-based sites (pairwise Fisher’s test: *P* = 0.065), likely because the sample size of the former was small (n = 7).

**Table 2:** Percent of students who observed contraceptive and abortion care topics during their Ob/Gyn clerkship, by religious affiliation of clerkship site.

	Overall responses (n)	Overall (%)	NFB (%)	C (%)	J (%)	P (%)	Pairwise fisher’s tests *P*-values*						
							*P*-value^#^	NFB-C	NFB-J	NFB-P	J-C	J-P	P–C
Patient counseling about methods of contraception (including the associated risks, benefits, and patient safety implications)	71	69.6	90.7	34.6	53.3	71.4	**0.000**	**0.000**	**0.008**	0.270	0.394	0.648	0.212
Prescription of appropriate contraception to patient(s)	64	62.7	79.6	34.6	46.7	71.4	**0.000**	**0.001**	0.061	0.634	0.620	0.572	0.212
Non-directive patient counseling about pregnancy termination options	26	25.5	35.2	7.7	20.0	28.6	**0.047**	0.079	0.532	1.000	0.532	1.000	0.532
Ultrasound (of a patient planning an abortion)	20	19.6	24.1	7.7	20.0	28.6	0.274	0.125	1.000	1.000	0.672	1.000	0.570
Discussion and prescription of medications for a non-surgical abortion	29	28.4	37.0	11.5	20.0	42.9	0.062	0.117	0.530	1.000	0.781	0.530	0.279
Surgical abortion procedure	29	28.4	35.2	15.4	33.3	14.3	0.257	0.672	1.000	0.818	0.744	0.924	1.000
Examination of products of conception	25	24.5	25.9	19.2	33.3	14.3	0.737	0.894	0.894	0.894	0.894	0.894	1.000
Follow up office visit for someone who recently had an abortion	29	28.4	42.6	11.5	13.3	14.3	**0.010**	**0.033**	0.198	0.458	1.000	1.000	1.000
None of the above	18	17.6	1.9	42.3	26.7	28.6	**0.000**	**0.000**	**0.021**	0.065	0.753	1.000	0.811

^#^*P*-value of Fisher’s exact test. *P-values of pairwise Fisher’s tests between religious affiliation of clerkship sites following family-wise error rate corrections. Bolded values represent those comparisons where *P* < 0.05. Abbreviations: NFB, non-faith-based; C, Catholic; J, Jewish; P, Protestant; n, sample size.

### Self-perceived competency in patient counseling

**Figure 2:**
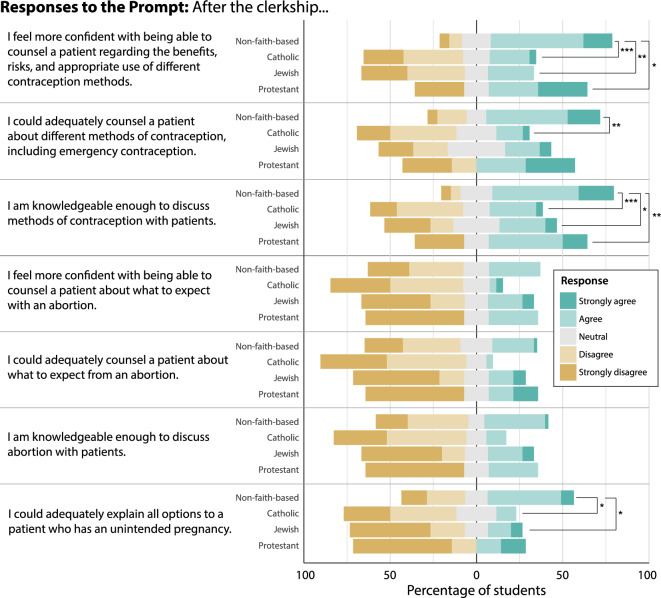
Results of self-assessed student competency in contraceptive and abortion counseling. All questions began with a prompt regarding their Ob/Gyn clerkship, “After the clerkship…”. All results are standardized by percentage of student responses within a religious affiliation category. Bars and asterisks represent a significant difference between religious affiliation of the clerkship site (pairwise Tukey differences resulting from linear model) at either the *P* < 0.05*, *P* < 0.01**, or *P* < 0.001*** level.

Irrespective of campus site or clerkship affiliation, the self-perceived competency of contraceptive counseling was higher than that of abortion counseling. Students felt they were most knowledgeable to discuss methods of contraception with patients (3.3 ± 1.3; Fig. 2, Table 3). Students were only slightly less confident in counseling patients regarding the benefits, risks, and appropriate use of contraception, and in counseling on different methods of contraception (3.2 ± 1.3, 3.1 ± 1.3, respectively). Self-perceived competency regarding topics of abortion care was low (Fig. 2, Table 3). Of abortion care topics, students were most competent in adequately explaining all options of an unintended pregnancy to patients (2.6 ± 1.3) and least competent in their confidence and ability to adequately explain to patients what to expect from an abortion (2.3 ± 1.2).

**Table 3:** Results of self-assessed student competency in contraceptive and abortion counseling following the Ob/Gyn clerkship, and perceived adequacy of training, by religious affiliation of clerkship site.

Survey question	Site	Overall			NFB			C			J			P		
		n	Mean	Std.	n	Mean	Std.	n	Mean	Std.	n	Mean	Std.	n	Mean	Std.
After the clerkship, I feel more confident with being able to counsel a patient regarding the benefits, risks, and appropriate use of different contraception methods	Both	102	3.2	1.3	54	3.7	1.0	26	2.5	1.2	15	2.4	1.2	7	3.3	1.7
	JB	20	3.9	1.2	9	4.3	0.5	4	4.0	0.8	–	–	–	7	3.3	1.7
	OW	82	3.0	1.2	45	3.6	1.1	22	2.2	1.1	15	2.4	1.2	–	–	–
After the clerkship, I could adequately counsel a patient about different methods of contraception, including emergency contraception	Both	101	3.1	1.3	53	3.6	1.2	26	2.5	1.1	15	2.7	1.2	7	3.1	1.8
	JB	20	3.7	1.3	9	4.1	0.9	4	3.5	1.3	–	–	*–*	7	3.1	1.8
	OW	81	3.0	1.2	44	3.5	1.2	22	2.3	1.0	15	2.7	1.2	–	–	–
After the clerkship, I am knowledgeable enough to discuss methods of contraception with patients	Both	102	3.3	1.2	54	3.7	1.0	26	2.7	1.2	15	2.7	1.3	7	3.1	1.6
	JB	20	4.0	1.3	9	4.7	0.5	4	3.8	1.3	–	–	–	7	3.1	1.6
	OW	82	3.1	1.2	45	3.6	1.0	22	2.5	1.1	15	2.7	1.3	–	–	–
After the clerkship, I feel more confident with being able to counsel a patient about what to expect with an abortion	Both	102	2.3	1.2	54	2.5	1.2	26	2.0	1.0	15	2.3	1.4	7	2.1	1.5
	JB	20	2.0	1.3	9	2.1	1.3	4	1.5	1.0	–	–	–	7	2.1	1.5
	OW	82	2.4	1.2	45	2.6	1.1	22	2.1	1.0	15	2.3	1.4	–	–	–
After the clerkship, I could adequately counsel a patient about what to expect from an abortion	Both	101	2.3	1.2	54	2.5	1.1	26	1.8	0.8	14	2.1	1.4	7	2.3	1.7
	JB	20	2.0	1.3	9	2.0	1.1	4	1.5	1.0	–	–	–	7	2.3	1.7
	OW	81	2.3	1.1	45	2.6	1.1	22	1.9	0.8	14	2.1	1.4	–	–	–
After the clerkship, I am knowledgeable enough to discuss abortion with patients	Both	102	2.4	1.2	54	2.7	1.2	26	2.0	1.0	15	2.3	1.4	7	2.1	1.5
	JB	20	2.1	1.3	9	2.2	1.4	4	1.5	1.0	–	–	–	7	2.1	1.5
	OW	2.5	1.2	1.2	45	2.8	1.2	22	2.1	0.9	15	2.3	1.4	–	–	–
After the clerkship, I could adequately explain all options to a patient who has an unintended pregnancy	Both	102	2.6	1.3	54	3.1	1.3	26	2.2	1.0	15	2.1	1.4	7	2.1	1.7
	JB	20	2.6	1.5	9	3.1	1.6	4	2.3	1.0	–	–	–	7	2.1	1.7
	OW	82	2.6	1.2	45	3.0	1.2	22	2.2	1.0	15	2.1	1.4	–	–	–
Clinical experience in counseling and prescribing contraception is important to offer during medical school	Both	102	4.6	0.7	54	4.8	0.5	26	4.4	0.9	15	4.5	0.9	7	4.4	1.1
	JB	20	4.8	0.7	9	4.9	0.3	4	5.0	0.0	–	–	–	7	4.4	1.1
	OW	82	4.6	0.7	45	4.7	0.5	22	4.3	0.9	15	4.5	0.9	–	–	–
My preclinical curriculum during first and second year adequately prepared me to counsel and educate patients about different methods of contraception	Both	102	3.0	1.1	54	3.1	1.1	26	3.1	1.1	15	2.5	0.9	7	2.9	1.5
	JB	20	3.3	1.3	9	3.4	1.3	4	3.8	1.3	–	–	–	7	2.9	1.5
	OW	82	2.9	1.1	45	3.1	1.1	22	3.0	1.1	15	2.5	0.9	–	–	–
Clinical experience in abortion care is important to offer during medical school	Both	102	4.5	0.8	54	4.6	0.7	26	4.3	0.8	15	4.3	1.1	7	4.6	0.5
	JB	20	4.5	0.8	9	4.3	1.0	4	4.5	0.6	–	–	–	7	4.6	0.5
	OW	82	4.5	0.8	45	4.7	0.6	22	4.3	0.8	15	4.3	1.1	–	–	–
My preclinical curriculum during first and second year adequately prepared me to counsel and educate patients about abortion	Both	102	2.3	1.1	54	2.3	0.9	26	2.4	1.2	15	2.1	1.1	7	2.0	1.4
	JB	20	2.4	1.1	9	2.2	0.8	4	3.3	1.0	–	–	–	7	2.0	1.4
	OW	82	2.3	1.0	45	2.3	1.0	22	2.3	1.2	15	2.1	1.1	–	–	–

NFB, non-faith-based; C, Catholic; J, Jewish; P, Protestant; n, sample size; Std., standard deviation; JB, Jonesboro, Arkansas site of NYIT COM; OW, Old Westbury, New York site of NYIT COM.

Among clerkship sites, significant differences between religious affiliations existed for all contraception statements, and for one of the abortion statements (Table 4). Students at non-faith-based clerkships sites were significantly more confident in their ability to counsel patients regarding the benefits, risks, and appropriate use of contraception compared to students at religiously affiliated sites (Linear Model [LM]: *P* < 0.05; Fig. 2, Table 4). They also reported being significantly more knowledgeable in terms of discussing methods of contraception compared to students at religiously affiliated sites (LM: *P* < 0.05). Students at non-faith-based clerkships rated their ability to counsel patients on the different methods of contraception significantly higher than students at Catholic-affiliated sites (LM: *P* < 0.05). Among abortion statements, students at non-faith-based sites reported higher competence in adequately explaining all options to a patient with an unintended pregnancy, compared to students at Catholic and Jewish clerkship sites (LM:* P* < 0.05). For only two questions was the factor of NYIT COM site (OW vs. JB) significant (Tables 3 and 4). Both were statements of contraception competency for which religious affiliation also had a significant effect. For both, students at the JB site felt 1.1–1.2 points more competent than students at the OW site. Nevertheless, the effect of religious affiliation across both sites was the same polarity (Table 3).

**Table 4:** Results of linear models for self-assessed student competency in contraceptive and abortion counseling following the Ob/Gyn clerkship, and perceived adequacy of training.

Survey question		Site difference	Pairwise Tukey difference between clerkship religious affiliations					
		OW− JB	NFB− C	NFB− J	NFB− P	J− C	J− P	P–C
After the clerkship, I feel more confident with being able to counsel a patient regarding the benefits, risks, and appropriate use of different contraception methods	Est	− 1.087	1.171	1.104	1.305	0.067	0.201	− 0.134
	Ste	0.332	0.261	0.324	0.520	0.359	0.601	0.544
	*P*− value	**0.001**	**0.000**	**0.003**	**0.027**	0.852	0.852	0.852
After the clerkship, I could adequately counsel a patient about different methods of contraception, including emergency contraception	Est	− 0.834	1.091	0.691	1.116	0.400	0.425	− 0.025
	Ste	0.356	0.281	0.348	0.556	0.384	0.643	0.582
	*P*− value	**0.021**	**0.001**	0.100	0.100	0.449	0.613	0.966
After the clerkship, I am knowledgeable enough to discuss methods of contraception with patients	Est	− 1.168	1.072	0.813	1.572	0.259	0.759	− 0.500
	Ste	0.330	0.260	0.323	0.517	0.357	0.598	0.542
	*P*− value	**0.001**	**0.000**	**0.027**	**0.009**	0.469	0.311	0.430
After the clerkship, I feel more confident with being able to counsel a patient about what to expect with an abortion	Est	0.505	0.506	0.251	− 0.064	0.256	− 0.315	0.570
	Ste	0.358	0.282	0.349	0.560	0.386	0.647	0.586
	*P*− value	0.161	0.451	0.753	0.909	0.753	0.753	0.753
After the clerkship, I could adequately counsel a patient about what to expect from an abortion	Est	0.527	0.699	0.445	− 0.225	0.254	− 0.669	0.924
	Ste	0.347	0.273	0.348	0.543	0.383	0.633	0.568
	*P*− value	1.322	0.072	0.408	0.680	0.610	0.440	0.323
After the clerkship, I am knowledgeable enough to discuss abortion with patients	Est	0.565	0.635	0.494	0.053	0.141	− 0.442	0.583
	Ste	0.361	0.284	0.352	0.565	0.390	0.653	0.591
	*P*− value	0.120	0.165	0.492	0.926	0.861	0.751	0.564
After the clerkship, I could adequately explain all options to a patient who has an unintended pregnancy	Est	− 0.067	0.862	0.911	0.969	− 0.049	0.058	− 0.106
	Ste	0.376	0.296	0.368	0.589	0.407	0.681	0.617
	*P*− value	0.859	**0.027**	**0.045**	0.207	0.933	0.933	0.933
Clinical experience in counseling and prescribing contraception is important to offer during medical school	Est	− 0.319	0.332	0.239	0.597	0.093	0.357	− 0.265
	Ste	0.219	0.172	0.214	0.343	0.237	0.396	0.359
	*P*− value	0.148	0.254	0.531	0.254	0.696	0.554	0.555
My preclinical curriculum during first and second year adequately prepared me to counsel and educate patients about different methods of contraception	Est	− 0.508	0.046	0.578	0.696	− 0.532	0.117	− 0.649
	Ste	0.340	0.267	0.332	0.532	0.367	0.615	0.557
	*P*− value	0.138	0.863	0.370	0.370	0.370	0.863	0.370
Clinical experience in abortion care is important to offer during medical school	Est	0.159	0.305	0.371	− 0.093	− 0.065	− 0.464	0.398
	Ste	0.236	0.186	0.231	0.369	0.255	0.427	0.387
	*P*− value	0.502	0.332	0.332	0.802	0.802	0.459	0.459
My preclinical curriculum during first and second year adequately prepared me to counsel and educate patients about abortion	Est	− 0.243	− 0.130	0.123	0.499	− 0.252	0.376	− 0.628
	Ste	0.324	0.255	0.316	0.507	0.350	0.587	0.531
	*P*− value	0.455	0.699	0.699	0.699	0.699	0.699	0.699

For linear models, the model estimate is that of the first factor compared to the second. For instance, the estimate value of site difference is OW compared to JB. *P*-values for site differences are the results of linear models. *P*-values shown between clerkship affiliations are for pairwise Tukey differences following family-wise error rate corrections from the linear models. Bolded values represent those comparisons where *P* < 0.05. Abbreviation: NFB, non-faith-based; C, Catholic; J, Jewish; P, Protestant; JB, Jonesboro, Arkansas site of NYIT COM; OW, Old Westbury, New York site of NYIT COM. Est, linear model estimate; Std., linear model standard error.

### Objective knowledge

Students scored higher on the contraception questions (1.8 ± 0.7 of 3) compared to the abortion questions (1.4 ± 1.0 of 5; Table 5). There were no significant differences between religious affiliation of the clerkship site, nor were there any differences between campus sites on either the contraception or abortion questions, nor on the total score. Overall, student performance on the objective knowledge assessment, especially the abortion questions, was poor, and no student received a full score.

**Table 5:** Results of linear models for objective knowledge questions on contraceptive and abortion care topics.

	Overall			NFB			C			J			P				Paisrwise Tukey difference between clerkship religious affiliations					
	n	Mean	Std.	n	Mean	Std.	n	Mean	Std.	n	Mean	Std.	n	Mean	Std.		NFB− C	NFB− J	NFB− P	J− C	J− P	P–C
Contraception questions (out of 3)	102	1.8	0.7	54	1.9	0.8	26	1.7	0.7	15	1.7	0.6	7	1.7	0.5	Est	0.118	0.082	0.322	0.037	0.240	− 0.204
																Ste	0.168	0.208	0.333	0.230	0.385	0.349
																*P*− value	0.835	0.835	0.835	0.874	0.835	0.835
Abortion questions (out of 5)	102	1.4	1.0	54	1.4	1.0	26	1.2	1.0	15	1.6	1.0	7	1.0	0.8	Est	0.284	− 0.238	0.857	0.522	1.095	− 0.572
																Ste	0.235	0.291	0.466	0.322	0.539	0.488
																*P*− value	0.293	0.416	0.208	0.216	0.208	0.293
Total (out of 8)	102	3.1	1.4	54	3.3	1.4	26	2.9	1.4	15	3.3	1.3	7	2.7	1.0	Est	0.403	− 0.090	1.179	0.492	1.269	− 0.776
																Ste	0.326	0.404	0.648	0.447	0.749	0.678
																*P*− value	0.328	0.825	0.281	0.328	0.281	0.328

Full question text and answers are provided in Supplementary Note 1. For linear models, the model estimate is that of the first factor compared to the second. *P*-values shown between clerkship affiliations are for pairwise Tukey differences following family-wise error rate corrections from the linear models. Abbreviation: NFB, non-faith-based; C, Catholic; J, Jewish; P, Protestant; JB, Jonesboro, Arkansas site of NYIT COM; OW, Old Westbury, New York site of NYIT COM. Est, linear model estimate; Std., linear model standard error.

### Perceived adequacy of training

Students overwhelmingly agreed that clinical experience in contraceptive and abortion care is important to offer in medical school (4.6 ± 0.7 and 4.5 ± 0.8, respectively; Table 3). Students were neutral as to whether the preclinical curriculum regarding contraception adequately prepared them for patient counseling (3.0 ± 1.1) and somewhat disagreed that their preclinical curriculum prepared them to counsel patients on abortion (2.3 ± 1.1). Students also overwhelming thought that NYIT COM should include more information about contraception and abortion during their first- and second-year curriculum (87% Yes, 6% No, 7% Don’t know/Unsure) and during the third-year core Ob/Gyn clerkship (81% Yes, 8% No, 8% Don’t know/Unsure). There were no significant differences between religious affiliation of clerkship site nor between campus location for any questions regarding the adequacy of training.

## Discussion

Overall, NYIT COM medical students felt more competent in areas of contraceptive care compared to abortion care. Religious affiliation of the clerkship site did affect Ob/Gyn clerkship training and outcomes. Students at faith-based sites tended to observe less contraceptive care compared to those at non-faith-based sites. Concomitantly, self-perceived competency in contraceptive counseling for students at faith-based sites was lower than that of students at non-faith-based sites. This shortfall was particularly apparent in students who attended Catholic-affiliated clerkship sites (Figs. 1 and 2). Clerkship religious affiliation had less of an effect on students’ observations of, and self-perceived competency in, abortion care. Overall competency in abortion-related topics was low across all clerkship sites. Though in some key areas of abortion care, students at non-faith-based sites still observed more and perceived themselves as more competent (Figs. 1 and 2). Despite overall low scores on the objective knowledge questions, students showed higher competency in contraception topics than in abortion topics. Our results suggest a shortfall in family planning training, particularly in contraceptive care at faith-based, and particularly Catholic, sites and in abortion care overall. Indeed, nearly half of students (42.3%) at Catholic-affiliated sites observed none of the listed contraception or abortion care topics during their Ob/Gyn clerkship compared to just 1.9% at non-faith-based sites. As expected, few differences in clerkship outcomes existed between campus sites. Our data also show that students desire more family planning training at both the preclinical and clinical level.

Objective outcomes of medical student training at clerkship sites of differing religious affiliation have only been documented in one prior study. Bringley et al.^11^ found that students who performed their clerkships at Catholic sites displayed significantly lower contraception Objective Structured Clinical Exam scores compared to peers at non-faith-based sites. This is consistent with the results regarding contraceptive care observations and competency herein.

Differences in family planning competency between faith-based and non-faith-based sites have also been seen at the residency level^12,14,15^. Residents who completed their training at faith-based hospitals were less satisfied with their training, and less competent, in contraceptive and abortion care than residents in non-faith-based programs^14,15^. Residents at Catholic programs also spent more time post-residency gaining the family planning training needed to meet the competency of their peers^14^. Catholic residency programs specifically, even when compared to other faith-based programs, also displayed poor abortion training. Nationwide, 47% of residency directors at Catholic programs reported poor abortion training compared to 0% at other faith-based residency locations; however all faith-based residencies reported adequate contraceptive training^12^. Thus the effect of religious affiliation on family planning training appears to exist, to varying extents, at all medical training levels.

Regardless of religious affiliation, the trend shown here that training in contraception care was more comprehensive than in abortion care appears to be consistent across many studies. This trend has been documented in opinions of family planning curriculum adequacy at both the medical school^2,6^ (and herein) and residency level^12^. Indeed nearly 17% of medical schools report no formal education regarding abortion in either preclinical education or third-year Ob/Gyn clerkships^2^.

When differences in outcomes between faith-based and non-faith-based clerkship sites existed, those sites affiliated with the Catholic church typically had the lowest outcomes. The likely explanation for this is that Catholic hospitals are expected to follow the ERDs which contain many restrictions and regulations on contraceptive and abortion care^7^. Although these directives are applied to all Catholic-affiliated hospitals and practices, previous research has shown inconsistencies in the adherence to these policies^14^. Indeed, that students witnessed certain types of abortion and contraceptive care at Catholic sites at all, support this previous finding. Although the ERDs and religiously based restrictions resulting from them likely explain the lower competency seen for students in some family planning topics at Catholic clerkship sites, it does not address why students at Jewish and Protestant sites also show lower competency in some areas. While less is known regarding policy at non-Catholic healthcare sites, religiously based restrictions on family planning care have been documented at both Protestant and Jewish health care facilities^8,9^. However, restrictions at Protestant and Jewish facilities appear to be less uniform and expansive than those at Catholic health care sites^8–10,12^. Indeed, this mirrors our data in that students at Jewish and Protestant clerkship sites observed, and had self-perceived competency in, contraceptive and abortion care at levels that are intermediate to students at Catholic and non-faith-based sites. Whether the somewhat less comprehensive training at these sites is a result of lower levels of restrictive policy compared to Catholic-affiliated sites, may be due to an unrelated effect such as the overall quality of the clerkship site (see ‘Strengths and Limitations’ below), or any other factor, remains to be explored. It should be noted however that differences between Jewish/Protestant sites and non-faith-based sites were less consistent than those between Catholic and non-faith-based sites. Regardless, non-faith-based hospitals have, for nearly all outputs, higher scores than faith-based hospitals.

Addressing the differences in Ob/Gyn clerkship outcomes shown here may require medical schools to implement supplemental training to meet curricular standards. Research has shown that supplemental didactive sessions dedicated to contraceptive and abortion care result in both increased knowledge of, and comfort discussing, these topics at both the medical student^16^ and resident level^17^. Implementation of supplemental family planning training outside of clerkship sites may also become more imperative in light of recent legal turmoil following the Supreme Courts 2022 Dobb’s decision^18^. Increasing numbers of state-level restrictions regarding contraception and abortion are likely to further impede medical student training^19^. Without additional mechanisms to ensure family planning competency, the differences in family planning training shown here are likely to become more pronounced.

While our results show differences in some clerkship outputs during medical school, it remains unknown how this shortfall in training may impact patient care. Ob/Gyn residents who reported dissatisfaction with their family planning training at faith-based programs reported delayed competency in many areas of family planning and a hesitancy to provide some family planning services post-residency compared to their peers at non-faith-based programs^14^. Thus poor medical student training in family planning topics may result in a hesitancy to provide these types of care in the future. Limiting exposure to family planning care may also inhibit development of other aspects of training as well. For instance, exposure to clinical abortion training has been shown to improve attitudes regarding abortion and increase the understanding of the psychosocial context of patients seeking abortions at both a resident and medical student level^20,21^. Arming medical students with a comprehensive knowledge of family planning not only allows them to better counsel and treat their patients on these topics, but also fosters a humanistic understanding of patients.

Some limitations exist in our study. First, there may also be a self-selection bias in who completed this survey based on clerkship experience or passion regarding family planning access and training. Indeed, women completed the survey at about twice the rate of men (Table 1). However, it is unlikely that this affected our findings given that gender was not a significant factor in any of linear models of self-perceived competency (see Methods). Second, this study has a somewhat low response rate at a single institution. However, data on the effect of faith-based clerkship affiliation on family planning training is extremely limited. Given the large population of students at NYIT COM, the absolute number of survey participants is on par with, or greater than, other studies looking at this effect^12,14^. However, the sample size of some individual categories is quite low (e.g. n = 7 for Protestant sites), limiting the conclusions which can be drawn from these smaller groups. In addition, though this study focuses on only one institution, NYIT COM is unique in having two campuses in different regions of the United States. At both campus sites, students perform their Ob/Gyn clerkships at multiple regional locations. Thus, our data represents 28 total clerkship sites (OW: 15; JB: 13). Our results are therefore somewhat robust across a variety of locations. This does however raise a subsequent limitation regarding the inconsistency in quality between clerkship sites. Some clerkship sites may simply be of higher quality than others, and currently we have no data with which to control for this effect. Only additional information on other medical schools and clerkship locations can help to resolve these limitations.

Medical students who train at faith-based clerkship sites, particularly Catholic sites, show lower self-perceived competency in, and observations of, contraceptive care compared to their peers at non-faith-based sites. Overall self-perceived competency in, and observations of, abortion care was low regardless of clerkship site, though in some areas of abortion care students at non-faith-based still showed higher outputs than peers at faith-based-sites. To provide comprehensive family planning care to future patients, additional training in contraception and abortion should be provided throughout medical school and is highly desired by medical students.

## Methods

### Study sample

Ethical approval was obtained by the NYIT Institution Review Board, who determined this project to be exempt (BHS-1839). A survey was created to assess three main outcomes following the third-year Ob/Gyn clerkship: 1) student observations regarding contraception and abortion training during the clerkship, 2) self-perceived competency in contraception and abortion counseling confidence, ability, and knowledge following the clerkship, and 3) objective knowledge about abortion and contraception, and perceived importance and adequacy of training in these topics. The survey instructed students that the term ‘abortion’ as used in our questions included both medical and surgical abortions, but not spontaneous abortions. This nomenclature will be utilized when describing our results. The survey was distributed through REDCap^22,23^ to all students in the NYIT COM classes of 2023 and 2024 at both the Old Westbury, New York (OW) and Jonesboro, Arkansas (JB) sites. At both sites third-year students rotated to one of a number of regional hospitals and/or outpatient clinics selected based on either a matching system (OW and JB) or through application to specific sites directly (OW). Both campus sites followed the same learning objectives and followed a common educational curriculum for the Ob/Gyn clerkship, including didactics and supplemental online modules, cases, and questions. One minor difference between sites was that at JB, some students performed their clerkship at outpatient clinics not associated with a hospital, whereas at OW (and for most students at JB) any outpatient clinic work was performed at clinics associated with their clerkship hospital. At the time of invitation, all students had completed their core third-year Ob/Gyn clerkship. Subjects provided informed consent prior to completing the survey and all procedures herein followed NYIT and all other relevant guidelines and regulations.

### Survey mechanism

Student observations during the clerkship were assessed using eight statements regarding a topic of care students may have witnessed during their Ob/Gyn clerkship. Two were specific to contraception training, six were specific to abortion training, and a ninth choice was “None of the above”. To assess self-perceived competency in patient counseling on contraception and abortion, students were asked seven Likert-scale questions (1, strongly disagree; 5, strongly agree) regarding their perceived abilities following the Ob/Gyn clerkship. Finally, objective knowledge of contraceptive and abortion care was assessed via eight multiple-choice questions (full question text and answer choices in Supplementary Note 1). All topics of care, statements, and questions regarding abortion were taken or adapted from Gardner et al.^24^, and those regarding contraception were developed using the APGO guidelines^1^. Students were asked an additional four Likert-style questions regarding their perspectives on the adequacy of the contraceptive and abortion training they received. Finally, students were asked two Yes/No-style questions regarding whether more family planning information should be included in their preclinical and clinical curriculum.

Students were additionally asked demographic data including their class year, gender, self-described racial identity, religion, medical school name and location, clerkship name and location, and the religious affiliation of their clerkship site, if known. Prior to data analysis, each response was checked, and the religious affiliation of the clerkship site verified, and corrected if necessary, by the researchers using public record information of the clerkship sites. This was done as students were not always aware if the clerkship site had a religious affiliation. Religious affiliation was coded as either: non-faith-based (NFB), Catholic (C), Jewish (J), or Protestant (P). Here, Protestant includes all Protestant denominational families, including Methodist and Baptist hospitals. This was done in part for practical reasons, as hospitals belonging to any Protestant religions were a minority of our data. Other religious affiliations were presented in the survey, as well as an option to specify an ‘Other’ religion, however, options beyond the four above yielded no responses.

### Statistical analysis

To first determine if students self-selected into faith-based clerkship sites based on either student religious identity or gender, Fisher’s exact tests were used to test for differences in student characteristics among clerkship site religious affiliation. For each characteristic (student religious identity and gender), four tests were run to determine if there were 1) differences in the student population between faith-based and non-faith-based sites for the whole sample, 2) differences in student characteristics among the four categories (NFB, C, J, P) of clerkship site for the whole sample, 3) or differences among the four categories (NFB, C, J, P) of clerkship site for each campus site individually.

Fisher’s exact tests were then used to test whether the number of students who observed a topic of contraceptive/abortion care differed between the four categories of religious affiliation. Post-hoc pairwise Fisher’s exact tests were then performed to investigate significant differences in each observational topic between specific religious affiliation categories. Linear models were used to compare outcomes between clerkship affiliations for the Likert-style questions as well as the objective knowledge questions. A linear model was constructed for each variable under consideration with religious affiliation set as a fixed effect. For these analyses, campus site was also included as a fixed effect. Significant differences between religious affiliations, and between sites, were determined using Tukey pairwise comparisons for linear models and family-wise error rates corrections^25^. For all Likert-scale questions, an additional linear model was constructed which also incorporated student religious identity and gender as factors. This was to determine if either of these had a significant effect on self-perceived competency or adequacy of training. However, in none of these models did student religious identity or gender have a significant effect, and in all cases the simpler model excluding these factors performed better as determined by lower Akaike Information Criterion values. Thus, only the simpler, better-performing models excluding these factors are presented. All data analysis was performed in R version 4.2.2^26^ with some tests utilizing the package ‘multcomp’^27^.

### Supplementary Information

Supplementary Information.

## Data Availability

The datasets generated and/or analyzed during the current study are available from the corresponding author on reasonable request.
