# Supplementary material for: Family planning competency following medical school Ob/Gyn clerkships at faith-based and secular sites
Source: Sci Rep. 2024 Feb 14;14:3667. doi: 10.1038/s41598-024-54304-5 (PMC10864354; doi:10.1038/s41598-024-54304-5)
Supplement: Supplementary file 1 — Supplementary Information. [file 41598_2024_54304_MOESM1_ESM.docx]

**Supplementary Information for:**

Family planning competency following medical school Ob/Gyn clerkships at faith-based and secular sites

Rachel N Feltman^1^, Steven R Lewis^2^, Nathan E Thompson^3,a,^*

^1^ *NYIT College of Osteopathic Medicine, Old Westbury, NY 11568 USA*

^2^ *Department of Clinical Medicine, NYIT College of Osteopathic Medicine, Jonesboro, AR 72401 USA*

^3^ *Department of Anatomy, NYIT College of Osteopathic Medicine, Old Westbury, NY 11568 USA*

^a^ ORCID ID: https://orcid.org/0000-0002-9273-3636

**Contents:**

Supplementary Table S1

Supplementary Note 1

**Supplementary Table S1**

**Table S1:** Association tests between characteristics of survey respondents and religious affiliation of the clerkship site.

| **Demographic variable** | **Clerkship variable** | **Fisher’s exact test P-value^#^** |
| --- | --- | --- |
| Student gender | Faith-based versus non-faith-based | 0.53 |
|  | Among categories (NFB, C, J, P) | 0.78 |
|  | Old Westbury site only | 1.00 |
|  | Jonesboro site only | 0.57 |
| Student religious affiliation | Faith-based versus non-faith-based | 0.13 |
|  | Among categories (NFB, C, J, P) | 0.09 |
|  | Old Westbury site only | 0.17 |
|  | Jonesboro site only | 0.79 |

Notes: ^#^P-value of Fisher’s exact test. Abbreviations: NFB, non-faith-based; C, Catholic; J, Jewish; P, Protestant; n, sample size. No test was significant, suggesting a lack of student self-selection into faith-based or non-faith-based clerkship site based on student religious affiliation or gender.

**Supplementary Note 1**

Below are the eight multiple choice questions used to assess objective knowledge. Questions 1–3 relate to contraceptive care and questions 4–8 relate to abortion care. The correct answer choices are those shown in bold.

1. Which of the following correctly shows the effectiveness of different methods of contraception from the most effective to least effective?
   1. **Long-acting reversible contraception (i.e. copper IUD, hormonal IUD, implant); Depo-Provera injection; estrogen/progesterone containing methods (i.e. pill, patch, or ring); barrier methods**
   2. Barrier methods; Depo-Provera injection; long-acting reversible contraception (i.e. copper IUD, hormonal IUD, implant); estrogen/progesterone containing methods (i.e. pill, patch, or ring)
   3. Estrogen/progesterone containing methods (i.e. pill, patch, or ring); barrier methods; Depo-Provera injection; long-acting reversible contraception (i.e. copper IUD, hormonal IUD, implant)
   4. Depo-Provera injection; long-acting reversible contraception (i.e. copper IUD, hormonal IUD, implant); barrier methods; estrogen/progesterone containing methods (i.e. pill, patch, or ring)
2. Methods of permanent female sterilization include: (select all that apply)
   1. **Tubal ligation**
   2. IUD insertion
   3. **Hysteroscopic tubal occlusion**
   4. Vasectomy
3. What types of contraception can be started immediately following a surgical abortion? (select all that apply)
   1. **Combined oral contraceptive pills (OCPs)**
   2. **Progestin only pills (POPs)**
   3. **NuvaRing (vaginal ring)**
   4. **Depo-Provera**
   5. **IUD (Mirena or Paragard)**
   6. **Implant (Nexplanon)**
4. What percentage of pregnancies in the United States are unintended?
   1. 30%
   2. 40%
   3. **50%**
   4. 60%
   5. 70%
5. Before a first trimester surgical abortion, standard of care is to complete which of the following: (select all that apply)
   1. Abdominal ultrasound
   2. **Transvaginal ultrasound**
   3. **Blood count (Hemoglobin/Hematocrit)**
   4. **Confirm Rh status**
   5. Type & Screen
   6. NPO status for 8 hours prior to procedure
6. After a first trimester surgical abortion, what steps are routinely taken to verify the procedure is complete?
   1. Ultrasound
   2. **Examination of the tissue (products of conception)**
   3. Tissue sent to pathology
   4. Serial measurement of beta hCG
7. Which of the following are true regarding complications of the first trimester surgical abortion: (select all that apply)
   1. **The most common complications following a first trimester abortion include need for reaspiration, incomplete abortion, bleeding, infection, and uterine perforation**
   2. First trimester abortion increases risk of ectopic pregnancy and/or miscarriage in future pregnancies
   3. **Risk of serious complication after first trimester abortion (including transfusion or hospital admission) is less than 1%**
   4. **Risk of death from first trimester surgical abortion is less than 1/1,000,000**
8. Which is the correct sequence for medication abortion?
   1. **One dose of Mifepristone then 24-72 hours later one dose of Misoprostol**
   2. One dose of Misoprostol then 24-72 hours later one dose of Mifepristone
   3. Two doses of Mifepristone separated by 24-72 hours
   4. Two doses of Misoprostol separated by 24/72 hours
